# Does the cortisol response to stress mediate the link between expressed emotion and oppositional behavior in Attention-Deficit/Hyperactivity-Disorder (ADHD)?

**DOI:** 10.1186/1744-9081-6-45

**Published:** 2010-07-15

**Authors:** Hanna Christiansen, Robert D Oades, Lamprini Psychogiou, Berthold P Hauffa, Edmund J Sonuga-Barke

**Affiliations:** 1 Clinic for Child & Adolescent Psychiatry and Psychotherapy, University of Duisburg-Essen, Germany; 2 Department of Clinical Psychology, Philipps-University Marburg, Germany; 3 Department of Psychiatry, University of Oxford, Oxford, UK; 4 University Children's Hospital, Dept. of Pediatric Endocrinology and Diabetes, Essen, Germany; 5 Developmental Brain-Behavior Unit, University of Southampton, Southampton, UK; 6 Ghent University, Dunantlaan, Ghent, Belgium; 7 MRC Social Genetic Developmental and Psychiatry Centre, Institute of Psychiatry, London, UK

## Abstract

**Background:**

Expressed Emotions (EE) are associated with oppositional behavior (OPB) in children with Attention Deficit/Hyperactivity Disorder (ADHD). EE has been linked to altered stress responses in some disorders, but ADHD has not been studied. We test the hypothesis that OPB in ADHD is mediated by altered stress-related cortisol reactivity to EE.

**Methods:**

Two groups of children (with/without ADHD) and their respective parents were randomly assigned to two different conditions with/without negative emotion and participated in an emotion provocation task. Parents' EE, their ratings of their children's OPB and their children's salivary cortisol levels were measured.

**Results:**

Low parental warmth was associated with OPB in ADHD. High levels of parental EE elicited a larger cortisol response. Stress-related cortisol reactivity mediated the EE-OPB link for all children. This highlights the general importance of parent-child interactions on externalizing behavior problems.

**Conclusion:**

High EE is a salient stressor for ADHD children that leads to increased levels of cortisol and OPB. The development of OPB might be mediated by the stress-response to high EE.

## Introduction

Expressed Emotion (EE; i.e., hostility, criticism, low warmth) directed by a caregiver towards a psychiatric patient, predicts relapse [1,2] and is associated with psychosis, depression [3,4], bipolar affective disorder [5] and a range of child and adolescent disorders [6,7]. For childhood and adolescence, there is a specific association between EE and Attention Deficit/Hyperactivity Disorder (ADHD), a chronic disorder with childhood onset marked by inattention, hyperactivity, and impulsiveness [8]. In cross-sectional as well as longitudinal studies, parents of ADHD children were more hostile, critical, and less warm [6,9-11]. Parental warmth on the other hand seems to be a protective factor. A decreased risk for the development of ADHD in low birth weight children was found when mothers showed high levels of warmth [12]. Taylor et al. [13] found that high EE increases the risk for the development of comorbid oppositional behaviour problems (OPB) in children with ADHD. Consistent with this view comorbid Oppositional Defiant Disorder (ODD) and Conduct Disorder (CD) in ADHD children is significantly predicted by mothers' and fathers' negative EE [14] with effects generalizing across national and cultural settings.

The mechanism that might link EE and OPB in children with ADHD remains unclear. Taylor [15] has suggested that child ADHD provokes EE and inefficient parental coping with both negative EE and poor coping contributing to the development of OPB. In the current study we explore the hypothesis that altered stress reactivity in children with ADHD might mediate the link between parental EE and childhood OPB. There are a number of features that point to such a hypothesis.

First, there is evidence, that hypothalamic-pituitary-adrenal (HPA) axis regulation is altered in children with ADHD. Low basal cortisol levels, weak acute HPA reactivity and abnormal diurnal cortisol rhythms have been reported [16-18] and specific influences of ADHD subtypes, such as inattention [19], on HPA responses. However, an interpretation of these results is complicated by studies reporting no differences in cortisol levels between children with and without ADHD [20] and others that describe a positive relationship between ADHD and cortisol levels [21,22].

Second, patients with OPB and ADHD may be particularly likely to show altered physiological reactivity. In a study by Freitag et al. [23] children with ADHD and ODD showed an attenuated cortisol awakening response compared to children with ADHD without ODD/CD and control children. Weaker cortisol responses in patients with ADHD and ODD were found compared to patients with ADHD only and normal controls [20]. Aggressive symptoms of CD were more clearly related to low cortisol concentrations than non-aggressive OPB in a study by Oosterlaan et al. [24]. Indeed, only a minority of ADHD patients with comorbid OPB showed significant cortisol increases in response to a psychological test in a study by Yang et al. [25], whereas behavioral stress responses and problems of aggression as measured with the Child Behavior Checklist (CBCL) correlated negatively for the majority of participants. In a recent study, disruptive behavior also predicted decreased cortisol reactivity in boys with ADHD, but only for the predominantly inattentive and hyperactive subtypes [26]. Other studies however, reported no differences between ADHD children with and without comorbid aggressive symptomatology [27,28], and longitudinal studies showed dependence of cortisol levels on current diagnostic status [17].

Third, EE may alter stress-related arousal patterns. Tarrier et al. [29] demonstrated that arousal of patients from low EE homes quickly habituated to the presence of their relatives, whereas arousal levels of patients with high EE relatives remained high [29,30]. Valone et al. [31] found that adolescents from high EE backgrounds had greater arousal, when anticipating an encounter with relatives, than did adolescents from low EE families. Hibbs et al. [32] found that in children with disruptive behavior disorders maternal EE was inversely related to spontaneous fluctuations in skin conductance response. A sensitization of neuroendocrine systems by repeated psychosocial stressors was reported in a study by Marcelis et al. [33] on psychotic patients. Vitaliano et al. [34] were able to show that among other variables EE explained physiological reactivity in response to an emotional task. Hooley et al. [35] examined fully remitted unipolar depressed participants and healthy control subjects with a novel, interpersonally based emotion provocation task with subjects hearing audiotapes of critical and praising remarks made about them by their own mothers. After being criticized by their mothers, fully remitted depressed participants still showed a greater increase in negative mood and a failure to activate the dorsolateral prefrontal cortex compared to healthy control subjects whereas no significant differences between controls and remitted depressed participants emerged in the positive control condition.

Despite these encouraging effects there has been no investigation of EE effects on HPA-axis activity and/or whether such effects might predict the development of OPB in ADHD children. Here we investigate this hypothesis making the following predictions: (1) EE in ADHD families is associated with oppositional behavior in the children with ADHD; (2) the cortisol response to a psychosocially stressful experimental condition is decreased for ADHD children with/without OPB, and this contrasts with the absence of differences between cases and controls in a positive control condition; (3) altered stress reactivity mediates the link between EE and the presence of oppositional behavior.

## Methods and materials

### Participants

Participants were 62 children with ADHD, their parents, and 61 healthy controls and their parents (see table 1). A power analysis with G-Power^© ^established that a total of 128 participants would be required to test for medium group effects when applying multivariate tests (F(1,126) = 3.91, Lambda = 13.33, α = 0.05, β = 0.95, f = 0.25); thus our sample missed the optimal sample size by five individuals. ADHD Children were recruited from a large outpatient clinic at the University Clinic for Child and Adolescent Psychiatry and Psychotherapy, Essen, Germany. The ADHD diagnosis was based on the Parental Account of Childhood Symptoms (PACS); a DSM-IV based, semi-structured, standardized, trained investigator-based (first author) interview that has good inter-rater reliability (r = 0.79 - 0.96; [36]). The PACS also screens for internalizing disorders such as anxiety and depression, and children screening positive were excluded from the study. Exclusion criteria for both cases and controls also included extreme over- and underweight, autism, epilepsy, general learning difficulties, brain disorders and any genetic or medical disorder associated with externalizing behavior that mimics ADHD. Additionally, parent/teacher ratings (Conners-Scales DSM-IV ratings ≥ 65) were used to assess oppositional behavior. While 37.1% of cases were on regular stimulant medication, all were off medication for 48 hours prior to participation. A study protocol in accord with the criteria of the Declaration of Helsinki was reviewed and approved by the local institutional review board and verbal and written consent was obtained from parents and children. Groups were matched for age and gender (see table 1 for sample characteristics). Children's age ranged from 5 to 17 years; parental age ranged from 29 to 52 years. All children had IQ > 70 (short version of the WISC; information, picture arrangement, similarities and block-design: [37]). There were more male children than females and more mothers than fathers.

**Table 1:** Characteristics of the sample (standard deviations and percentages in parentheses)

	N	Age	Males	IQ
**Cases with ADHD**	62	10.6 (2.8)	50 (80.6%)	102.8 (14.4)
**Control children**	61	10.5 (2.7)	49 (80.3%)	110.6 (12.6)
**Parents of cases with ADHD**	62	39.2 (4.6)	3 (4.8%)	not assessed
**Parents of control children**	61	41.2 (5.1)	5 (8.2%)	not assessed

### Measures

**ADHD symptoms **and **oppositional scores **were rated with the long version of the Conners' parent and teacher rating scales (CPRS-R:L; CTRS-R:L; [38]). The questionnaires assess symptoms on 14 scales including DSM-IV based ADHD scales (based on 9 inattention and 9 hyperactive/impulsive symptoms), and oppositional behavior [39]. T-scores were calculated for all scales [38] and missing subscale data were prorated if 7 or more from 9 items were present.

**Expressed emotion **was measured using the Five Minute Speech Sample (FMSS: [40]; for the German adaptation see [41]). A trained interviewer (first author) asked the parent accompanying the child to talk for five minutes about the child prior to the PACS interview. Ratings, based on the number of critical and/or positive comments, as well as tone, were made for the following five categories: initial statement, high/low EE, emotional over involvement (EOI), warmth, and hostility. The tapes were coded by two independent raters (inter-rater agreement Cohen's Kappa = .83; average agreement of 97.2%). Additionally, all children were asked how critical their mum/dad is of them generally (perceived criticism/PC: [42]), a measure that correlates highly with EE outcomes [42,43].

### Social stress reactivity

#### Induction of social stress

We used the novel experimental attitude priming task (APT) described by Hooley et al. [35], to create a psychosocially stressful situation, designed to affect cortisol responses. Before the APT, all children made a rating of perceived criticism (PC, baseline measure) in the absence of their parents. Then parents were asked to say either three positive (control condition) or three negative (experimental condition) things about their child with the child listening (face to face). Negative comments for both experimental and control group were typically things like *"he/she never cleans up his room"*; *"I don't like him/her quarreling with his siblings"*; positive comments were i.e. *"he/she is a friendly person"*, *"he/she is helpful"*. To further enhance the emotional provocation, parents either praised (positive condition) all the hits the child made on a short stop-signal computer task [44,45] or criticized all errors (negative condition). After this task the child gave a final PC rating.

#### Cortisol stress response

Saliva samples of cortisol were used as a marker for the acute response to stress in both parents and children. Over the period of one hour, five samples were taken. The first one (T1) served as baseline measure. The second one (T2) was taken while the child worked on the computer (during stress task); the third one (T3) was taken immediately after the stress task; T4 and T5 were taken 30 and 60 minutes after the baseline measures, that is 15 respectively 30 minutes after the stress task. For the detection of un-elicited cortisol response, saliva samples were subjected to standard techniques. Briefly, participants were asked to place a cotton wool swab into their mouth for two minutes. The swab was subsequently placed into a prepared tube, which was stored at room temperature until collection of all five samples. Tubes were then centrifuged for 10 min and stored at -20°C prior to analysis. Samples were inspected for blood contamination before further analysis. All samples yielded clear saliva of low viscosity. Saliva flow was determined by weighing the tubes before and after saliva sampling. Cortisol concentration in saliva was measured using a commercially available, coated-tube radioimmunoassay validated for that matrix [46]. Measurements were performed in duplicate. Intra- and inter-assay variability (CV %), as calculated by the Rodbard method [47], were 5.35 and 12.24 at a physiologic concentration range (0.28 - 0.52 μg/dL), 2.09 and 3.67 at a high concentration range (0.798 - 1.30 μg/dL), and 1.64 and 2.92 at very high concentrations (2.06 - 3.36 μg/dL). Acute and chronic health problems, regular medication, current stress, time of getting up and breakfast were controlled to avoid contamination of the saliva samples. All participants were asked not to eat or drink (except water) during the experiment.

### Procedure

All children and their parents arrived at 8 a.m. to ensure that no activities except breakfast and the trip to the clinic occurred that could influence early morning free cortisol levels [48,49]. After a relaxation exercise the baseline cortisol sample was taken (T1) at 9 a.m. Participants were randomly divided into two subgroups according to the experimental conditions described above and then performed a ten minute stop-signal task during and after which four further measures of salivary cortisol were taken over a one hour period (till 10 a.m.).

### Statistical analyses

Data reduction and analyses were carried out using the statistical package SPSS 15.0. Differences in Conners' rating scales and EE measures between ADHD children and controls were compared with an ANOVA. Additionally oppositionality ratings were entered as covariate to control possible influences on EE measures, since comorbid OPB strongly influences the association between ADHD and EE [13,14]. Influence of medication on cortisol response, CPRS-R/CTRS-R-ratings and FMSS-ratings were explored using Pearson correlations. Correlations between FMSS measures and PC were calculated to validate those two EE measures. Differences in PC ratings over time and groups were tested with a MANOVA with repeated measures. To test the hypothesis, whether EE in ADHD is associated with OPB, linear regressions were used to predict comorbid oppositionality ratings with FMSS measures. To compare the psychosocial stress reactivity in cases and controls, baseline cortisol levels were first tested for equivalence in both groups (ANOVA). This was followed by a MANOVA with repeated measures to test the three way interaction between time × condition (positive/negative) × group (cases/controls). OPB was entered as a covariate to control for its possible influence.

To establish a mediation effect, first the predictor-criterion correlation must be significant and meaningful. Second, the predictor must show a significant association with the putative mediator. Third, the mediator must be associated with the criterion measure, and finally, the predictor-criterion correlation must be attenuated when the mediator variable is entered into the mix [50,51]. Thus according to our hypothesis, regression analyses must show that a measure of EE is significantly correlated with oppositional behavior and also with cortisol response. The mediator cortisol response must be correlated with oppositional behavior and finally, the link between EE and oppositional behavior should be reduced if cortisol response is included in the regression. This model was then tested with the Sobel-test.

## Results

### Preliminary analyses

Children with ADHD scored higher than control children on all Conners' subscales, with T-scores ranging between 1.5 to 2 standard deviations above the mean, except for parental anxiety ratings (see table 2). There were only six ADHD children with both parent and teacher oppositionality scores within the normal range. Due to randomization four of them were in the positive and two in the negative condition. Thus our sample consisted mostly of children with comorbid OPB.

**Table 2:** Mean ratings on the Conners' subscales and Expressed Emotion (EE) Measures for ADHD and control children with SD in parenthesis and ANOVA results on significant differences.

Conners subscale	ADHD	Controls	ANOVA	ADHD	Control	ANOVA
	parent ratings			teacher ratings		
***oppositional***	75.92 (10.66)	51.44 (7.64)	F_(1,121) _= 213.34, p < .001	67.67 (14.01)	48.93 (5.43)	F_(1,114) _= 87.72, p < .001
***inattention***	72.47 (10.12)	48.41 (5.78)	F_(1,121) _= 260.56, p < .001	63.58 (8.96)	44.66 (12.96)	F_(1,114) _= 84.56, p < .001
***hyperactivity***	78.48 (10.13)	47.84 (5.81)	F_(1,121) _= 421.45, p < .001	73.58 (10.91)	46.46 (3.97)	F_(1,114) _= 307.77, p < .001
***anxious-shy***	57.95 (13.81)	49.43 (8.82)	F_(1,121) _= 16.58, p < .001	64.6 (11.55)	53.57 (9.34)	F_(1,114) _= 31.67, p < .001
***social problems***	69.73 (14.75)	50.46 (7.65)	F_(1,121) _= 111.01, p < .001	62.23 (13.80)	47.89 (6.88)	F_(1,114) _= 49.0, p < .001
***emotional lability***	66.60 (13.50)	47.21 (7.95)	F_(1,121) _= 92.77, p < .001	68.65 (13.62)	47.68 (6.31)	F_(1,114) _= 95.42, p < .001
***DSM-IV-ct***	78.56 (8.56)	47.33 (4.84)	F_(1,121) _= 616.68, p < .001	73.75 (8.54)	47.46 (6.10)	F_(1,114) _= 359.0, p < .001
**parental EE-rating**			**ANOVA**		**Effect size *d***	
***negative comments***	5.46 (3.84)	2.60 (2.93)	F_(1,117) _= 13.72, p < .001		.84	
***positive comments***	5.58 (2.93)	8.16 (2.08)	F_(1,117) _= 19.06, p < .001		-1.01	
***warmth***	2.75 (1.38)	3.79 (1.08)	F_(1,117) _= 14.46, p < .001		-.84	
***criticism/hostility***	2.44 (1.79)	0.84 (1.13)	F_(1,117) _= 22.55, p < .001		1.07	

Effect sizes are reported for EE ratings only.

There were no influences of medication status on any of the measures taken. Neither cortisol levels (μg/dL) at baseline (*r *-.011, p = .469) nor on the following four measures (T2*r *.110, p = .226; T3*r *.045, p = .379; T4*r *-.005, p = .486; T5*r *-.099, p = .247) nor Conners' parent (*r *.081, p = .283) or teacher (*r *.06, p = .340) ratings of the DSM-IV ADHD-combined type correlated significantly with medication status. There were no differences in FMSS ratings for children with or without medication (*F*(1,50) = .049, p = .826). For ADHD children Perceived Criticism (PC) did not correlate significantly with the assigned condition nor with parental FMSS ratings, suggesting that they did not perceive the criticism expressed by their parents. For the control children, on the other hand, there were significant correlations between assigned condition and PC at both times (see table 3).

**Table 3:** Correlations between Perceived Criticism (PC)-ratings (baseline/follow-up), Five Minute Speech Sample (FMSS) ratings, and experimental condition (p-values in parentheses).

		Perceived Criticism baseline measure	Perceived Criticism final measure
Children with ADHD	Condition (positive/negative)	-.150 (.246)	-.151 (.241)
	FMSS hostility rating	+.248 (.060)	+.237 (.073)
	FMSS warmth rating	-.067 (.617)	-.104 (.437)
	Perceived Criticism baseline	1	+.840**(.001)
Control Children	Condition (positive/negative)	+.280* (.029)	+.261* (.042)
	FMSS hostility rating	+.191 (.148)	+.190 (.150)
	FMSS warmth rating	-.312* (.016)	-.298* (.022)
	Perceived Criticism baseline	1	+.934**(.001)

### EE in ADHD is associated with oppositional behavior

Fifty nine tapes from parents of ADHD children, and 58 tapes from parents of control children could be scored. An ANOVA revealed significant differences in EE ratings with parents of ADHD children showing more EE than those of control parents (Mean of Squares = 17.527, *F*(1,116) = 13.422, p < .001, η^2 ^= .105; for details in FMSS-ratings see table 2). When entered as covariate, OPB ratings removed significant differences (*F*(1,107) = 0.008, p = .929), but age did not (*F*(6,109) = 8.38, p < .001, η^2 ^= .316). Of the parents with ADHD children, 28.6% were rated with High EE, 6.3% showed EOI, and 4.8% showed both High EE and EOI, whereas 54% showed Low EE. In comparison, parents of control children showed mostly Low EE (83.3%); 5% were highly critical and 8.3% showed EOI. Effects were due to higher criticism/hostility (*d *1.07) and reduced warmth (*d *-.84) in parents of ADHD children, but not to EOI. A stepwise linear regression with all FMSS measures was used to predict oppositionality in cases and controls. A significant prediction could only be obtained for ADHD children with the EE sub-measure of high warmth predicting low oppositionality ratings (*R *= .271, *F*(1,56) = 4.45, p = .039).

### Cortisol response to a psychosocially stressful experimental condition is decreased for ADHD children, but no differences between cases and controls are expected for the positive control condition

**Figure 1:**
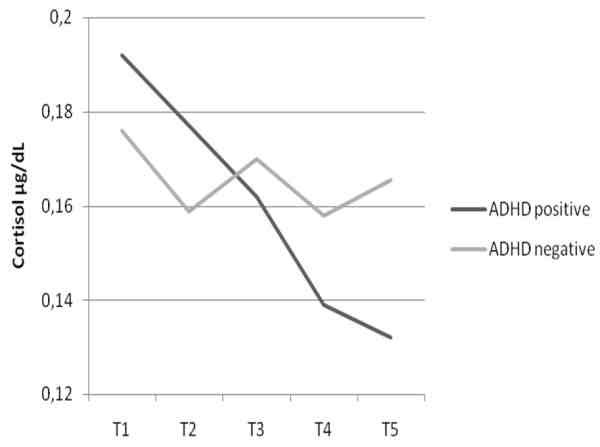
**Distribution of Cortisol means for children with ADHD according to emotion provocation at five time points across 60 minutes (see text for details)**. Positive: positive priming + parental reinforcement for hits during computer-task. Negative: negative priming + parental criticism for errors during computer-task.

**Figure 2:**
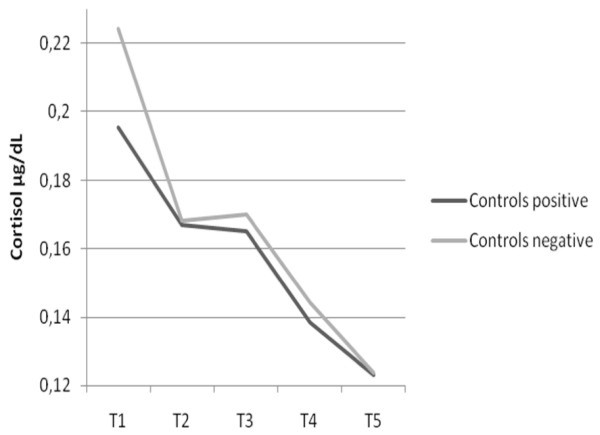
**Distribution of Cortisol means for control children according to emotion provocation at five time points across 60 minutes (see text for details)**. MANOVA: main effect cortisol: F(4,220) = 27.14, p = .000, η^2 ^= .33 Interaction cortisol × condition × group: F(16,892) = 2.04, p = .009, η^2 ^= .035. Cohens *d *comparing T5 for ADHD positive/negative = .40. Cohens *d *comparing T5 for Controls positive/negative = .008. Cohens *d *comparing T5 for ADHD positive with Controls positive = .15. Cohens *d *comparing T5 for ADHD negative with Controls negative = .50.

First baseline cortisol levels (T1) were compared that revealed no differences between cases and controls: ADHD: mean = .182, SD = .098; controls: mean = .210, SD = .117; *F*(1,120) = 2.14, p = .146. Thus no general differences between ADHD and control children in cortisol level at baseline needed to be taken into account in the analysis. A multivariate analysis with repeated measures compared cortisol responses over time for both groups of children and the two conditions (negative vs. positive). There was a significant three way interaction (time × condition × group: *F*(16,892) = 2.04, p = .009, η^2 ^= .035). Adding oppositional ratings as a covariate removed the significant difference in cortisol response (*F*(4,219) = .479, p = .751, η^2 ^= .009), whereas controlling for age (*F*(4,105) = 2.70, p = .034, η^2 ^= .093) and gender (F(1,119) = 0.226, p = .0635, η^2 ^= .001) did not. As can be seen in figure 1 &2, control children showed the expected decrease over time without marked differences according to the two conditions (see figure 2) whereas the effects were different for ADHD children (see figure 1). Contrary to our expectation, ADHD children in the negative condition did not show a decrease in cortisol levels. Instead, there was an increase of cortisol after negative emotion provocation. However, ADHD children in the positive condition showed a decrease in cortisol response comparable to the control group.

### Altered stress reactivity, as indicated by altered cortisol responses, mediates the link between EE and the presence of oppositional behavior

**Figure 3:**
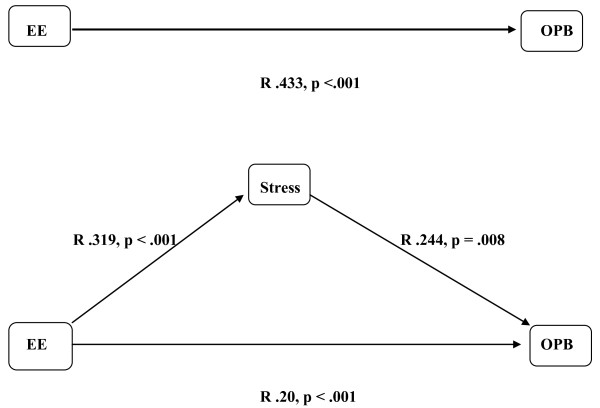
**Mediator-Model, with the independent variable Expressed Emotion (EE), the dependent variable oppositional behavior (OPB), and the cortisol stress index (Stress) as mediator variable**. The correlations between each element of the model are shown for all children.

To test the mediator model, Conners' ratings of oppositional behavior were used as a measure of OPB (see [39] for justification of this procedure). Parent and teacher oppositional ratings from the Conner's questionnaires were collapsed into one measure to avoid bias in either direction. All children were included in this model, since separate preliminary analyses for ADHD cases and controls failed to show significant relations between paths. Multiple linear regressions with all FMSS measures predicting oppositional behavior were calculated. The sub-measure positive comments emerged as a significant predictor and the direct path between this EE measure and oppositional behavior resulted in a significant medium correlation (*R *.433, p < .001). A stress-index that reflects decreases in cortisol levels from baseline (T1) to the final measure (T5) was used as the mediator variable. This was based on cortisol responses in the control group, since they showed the expected circadian decrease in cortisol response across the day with levels decreasing by 86.5% from T1 to T5 averaged over both conditions. Relative to this decreases in cortisol levels for ADHD children differed according to condition with children in the positive condition showing a decrease of 69%, and children in the negative condition one of only 12.7%. Figure 3 shows the mediator model to be tested.

For all children the path between EE (positive comments) and cortisol stress index (putative mediator) was significant (*R *.319, p < .001), as was the path between the putative mediator (cortisol stress response) and the dependent variable (conduct problems) (*R *.244, p = .008). The significant path between positive comments and OPB had already been established. Together these associations established pre-requirements for testing mediation (Baron & Kenny, 1986). This was achieved in a second step when the significance of the path between EE and OPB became reduced in significance when the putative mediator, stress response index was added to the regression model (*R *.20, p < .001). A Sobel-test was significant (Sobel-test = 2.926, p < .001; ta = 3.61, tb = -4.98) confirming partial mediation of the link between EE and OPB by stress response.

## Discussion

The study showed that parents of children with ADHD are more hostile and critical towards their children than parents of healthy controls. These results were significantly influenced by childrens' OPB, since oppositionality ratings entered as a covariate removed this significant effect. This confirms Taylor's [15] hypothesis that comorbid OPB contributes to the emergence of high EE in parents of ADHD. Indeed, in an earlier study, mothers of ADHD children who had responded positively to methylphenidate treatment, their expression of warmth increased and expressed criticism towards their child decreased [52]. Further, in a recent study by Sonuga-Barke et al. [14], ADHD children whose parents were highly critical and lacked warmth had significantly higher levels of ODD and CD. This study also found gene-environment interactions involving EE and polymorphisms of the serotonin and dopamine transporters suggesting that maternal expressions of warmth and hostility may act together with genetic factors in altering the severity of ADHD [53].

Contrary to our expectations, high criticism did not predict high oppositionality ratings but high warmth predicted low scores instead. This result was specific for the ADHD group and highlights the potentially protective role of parental warmth. For instance, Tully et al. [12] found that enhanced maternal warmth was predictive for low parent and teacher ADHD ratings, and Caspi et al. [54] showed that the monozygotic twin who received more maternal negativity and less warmth had more antisocial behavior problems compared to the twin receiving more warmth.

There were no significant differences in baseline cortisol between cases and controls. We were not able to confirm our hypothesis that ADHD children would show lower cortisol levels in response to a psychosocial stressor. This decrease of cortisol levels over the assessment period was absent in the ADHD children (negative condition). Control children and ADHD children in the positive control condition on the other hand did show a decrease in cortisol over time as did control children in the negative condition. This is in line with the findings by Hooley et al. [35] who only found effects of maternal criticism on remitted depressed patients. The majority of studies on ADHD children with comorbid OPB found decreases in cortisol levels [20,55], though these results are not entirely consistent. There were only six children in our study with Conner oppositional ratings below T-scores of 60 in both parent and teacher questionnaires, thus the majority of children in our study presented comorbid OPB within the clinical range. Severe oppositional problems predicted weaker adrenocortical reactivity for children with the predominantly inattentive and hyperactive, but not for the combined ADHD subtype [26]. Combined subtype ratings on the Conners' parent and teacher scales were overall high in our sample which might explain the discrepancy in findings. Also stress tasks in the studies mentioned were not based on parental EE. It might be that parental criticism is a highly salient psychosocial stressor for subjects with psychiatric disorders. The participants in the study by Hooley et al. [35] were fully remitted for a minimum of five months (range five to 109 months) and still reacted strongly to maternal criticism. There is also evidence that early disruptions in the parent-child-relationship, as is often found in families with ADHD children, produces increased cortisol levels in preschoolers. Further these heightened levels of cortisol predict increased behavioral and emotional problems in the school-aged child [56].

Family adversity, which can accompany ADHD, is related to higher and less-regulated cortisol activity in school-aged children and adolescents [57,58]. Children with behavioral problems have shown an increased cortisol response during a parent-child conflict-discussion task and were associated with dysfunctional parenting [59]. Typically, children with the largest increases in cortisol were rated as less capable of regulating negative emotions and aggression [60,61]. An impairment in the regulation of emotion, as part of the core symptom of impulsivity, is often observed in children with ADHD [62]. Effects of emotional states on saliva cortisol responses could also be demonstrated for situations encountered in daily life [63,64]. In both studies, emotions of loneliness, sadness, and anger, as assessed with diary reports, were associated with specific cortisol patterns. Depressive symptoms led to a decrease of basal cortisol levels and higher cortisol levels were observed for moments adolescents were alone rather than with others, and for trait anger. Responses to the negative condition by ADHD children suggest an emotional stress response maybe similar to feelings of anger/annoyance. ADHD children did perceive the criticism expressed by their parents, but this was independent of the experimental condition, since no significant correlations between the final perceived criticism rating and condition could be obtained (see table 3) for ADHD children. Thus, apparently, children with ADHD do not explicitly perceive an acute emotional stressor, but rather respond to it on a physiological level, whereas control children do show an explicit reaction to the priming condition (see table 3), but this is not reflected in a physiological response.

In agreement with our mediation hypothesis (see above), we demonstrated that positive EE were significantly linked to the psychosocial stress response which in turn attenuated the direct path between EE and oppositionality. Our results support the important role of positive/warm interactions between parent and child and its effect on oppositional behavior. Since we were able to show this for cases and controls, positive parenting might be a generally protective parenting factor. However, it would be very interesting to replicate findings in a larger sample with separate analyses for ADHD cases and healthy controls. High psychosocial stress on the other hand, as shown experimentally, results in increases of the cortisol response and the tension felt. This in turn might lead to feelings of stress/anger that are expressed in oppositional behavior as described in behavioral models on ADHD and comorbid OPB [65]. Since the emotion provocation task seems to be very useful in manipulating EE response, future studies should further explore the association of ADHD and OPB with respect to high EE.

### Limitations to the study

The power analysis prior to testing showed that our sample lacked five participants for optimal sample size. A post-hoc power analysis with the given sample size and 5 repeated measures between 2 groups established a satisfying power of .94 with Lambda = 12.81, F(1,121) = 3.81, α = .05, f = 0.25. Thus interpretation of results should not be overly biased.

For ethical reasons, we were not able to obtain blood samples from the participating families. Due to this, we were unable to assess the critical ACTH component of the HPA-axis. We also did not measure other stress variables such as heart rate or skin conductance response that could have confirmed the cortisol stress responses.

Since children with ADHD significantly differed from healthy control children in the Conners' subscale anxious-shy, possible influences on cortisol response had to be taken into account, even though children with positive screenings of internalizing disorders were not included in the study. We collapsed the parent and teacher ratings of the Conners' subscale anxious-shy into one measure and entered this as a covariate in an ANOVA with baseline cortisol as the dependent variable. Anxiousness did not influence levels of baseline cortisol significantly (F(1,112) = 0.100, p .752), though assessment of comorbid disorders on HPA-axis should be addressed in future studies on this topic to further confirm this result.

The sample in this study consisted of males and females, though it was predominantly male, representing the four to one prevalence of males to females in populations at large [66-68]. Gender did not affect levels in cortisol in our study, but a replication of these results in a sample of ADHD girls is recommended.

The age range in this sample was fairly large. It is well known that ADHD symptoms change over time, and for children reaching adolescence a reduction in impulsive/hyperactive behavior is often found [69]. But when entered as a covariate, age did not significantly influence our results.

Oppositional behavior was solely assessed with the Conners Rating Scales. A more thorough assessment of this comorbid condition, especially with respect to the differentiation between ODD and CD might contribute to more specific results.

EE were assessed with the short FMSS and not the original Camberwell Family Interview (CFI). Assessment of EE with the CFI takes approximately one to two hours followed by three to four hours rating. Assessment and rating of EE with the FMSS is much faster and correspondence between CFI-EE ratings and those from the FMSS have proven to be highly satisfactory [40], that the use of this measure seemed justified.

Positive and negative comments were not subjected to qualitative analysis. But since cortisol response of the ADHD children in the positive condition was so akin to that of the controls, we were able to show that ADHD children and controls respond similar to positive feedback and differently to negative feedback. The qualitative difference in negative feedback, that is highly likely, is reflected in the significant differences in high EE ratings by ADHD parents.

The cross-sectional study design did not allow us to determine the direction of effects. Future studies should report path models that show the structural relations between the variables analysed.

### Clinical implications

As shown in this and several other studies, parental high EE is a highly salient psychosocial stressor and proves to be relevant for comorbid OPB in ADHD patients. Approaches focusing on the reduction of parental hostility and the enhancement of warmth should be a target for therapeutic interventions to reduce or even prevent comorbid disorders such as OPB. Even though most behavior therapy programs include sessions on positive parent-child interactions their premises need to be further explored and might lead to an improvement of behavioral therapy elements. Furthermore, cortisol levels might serve as a relevant indicator for therapeutic success. Children with disruptive behaviors and low basal cortisol levels had a better response to intervention by parent training as assessed by changes in cortisol levels and disruptive behavior scores [70].

## Abbreviations

ADHD: Attention-deficit/hyperactivity disorder; OPB: Oppositional Behavior; CFI: Camberwell Family Interview; CP/TRS: Conners parent/teacher rating scale; DSM-IV, OPB: Conduct Problems; Diagnostic and statistical Manual of the American Psychiatric Association, 4^th ^version; EE: Expressed Emotion; FMSS: Five Minute Speech Sample; HPA: hypothalamo-pituitary-adrenal axis; ICD-10: International Classification of Diseases, 10^th ^version; ODD: Oppositional Defiant Disorder; PC: Perceived Criticism;

## Competing interests

The authors declare that they have no competing interests.

## Authors' contributions

All authors have read and approved the final manuscript. HC planned the study, analyzed the data, and wrote the manuscript. RDO, EJSB, and LP co-read the manuscript, and also wrote sections of it, and contributed analyzing strategies. BPH organized the cortisol analyses and advised on their interpretation 

## Authors' information

This work was partly funded by NIMH Grant RO 1MH062873 to S.V. Faraone and by Sarstedt, who supplied the Salivettes for the study. The NIMH and Sarstedt had no further role in the study design; in the collection, analysis and interpretation of data or in the writing of the report.
